# Plant-derived extracellular vesicles: an alternative and complementary therapeutic approach for genitourinary tumors

**DOI:** 10.3389/fcell.2025.1695636

**Published:** 2025-11-07

**Authors:** Yuqing Huang, Yunmeng Zhang, Kecheng Lou, Shangzhi Feng, Guoqiang Feng

**Affiliations:** 1 Department of Urology, Jiujiang University Clinic College/Hospital, Jiujiang, Jiangxi, China; 2 Department of Anesthesiology, Jiujiang University Clinic College/Hospital, Jiujiang, Jiangxi, China; 3 Department of Urology, Lanxi People’s Hospital, Jinhua, Zhejiang, China; 4 Department of Rehabilitation, Jiujiang University Clinic College/Hospital, Jiujiang, Jiangxi, China

**Keywords:** plant-derived extracellular vesicles, genitourinary tumors, tumor drug resistance, adjuvant anticancer drugs, engineered extracellular vesicles

## Abstract

Over the past four decades, the incidence of genitourinary tumors has risen significantly, becoming a major global health concern. This trend necessitates the exploration of more effective treatment strategies. Although considerable progress has been made in treatments such as endocrine, immune, and targeted therapies, these malignancies often remain uncontrolled, especially when metastatic. Recent advances in nanobiomedicine have revealed plant-derived extracellular vesicles as a novel and promising anti-cancer therapeutic option. These natural nanoparticles exhibit high biocompatibility, targeted delivery capability, and renewable properties, positioning them at the forefront of innovative cancer treatment strategies. Their inherent anti-cancer properties—such as inducing cell cycle arrest, promoting apoptosis, and inhibiting metastasis—may offer new alternatives for treating genitourinary cancers. This review summarizes current research on plant-based treatments for genitourinary tumors, focusing on the potential mechanisms and applications of plant-derived extracellular vesicles, thereby suggesting new nanoscale alternative and complementary therapeutic avenues.

## Introduction

Genitourinary cancers include malignancies of the urinary and reproductive systems, such as renal cancer, urothelial carcinoma (including renal pelvic, ureteral, and bladder cancers), prostate cancer, and germ cell tumors. Among these, kidney, bladder, and prostate cancers are the most common. With improving living standards, the incidence of these cancers has increased markedly (Tian et al., 2023). Despite diagnostic and therapeutic advances—e.g., PSA screening and endocrine therapy for prostate cancer, and immunotherapy for urothelial carcinoma (Jackson-Spence et al., 2023)—the unique pathogenesis of each cancer type complicates standardized treatment. A lack of understanding of these mechanisms hinders progress in precision medicine. Although targeted therapies have been developed, many exhibit limited clinical efficacy or significant side effects (Frederickson and Ylä-Herttuala, 2019; Bedard et al., 2020; Ederhy et al., 2011). This suggests that cancer may arise from an imbalance in bodily systems, a concept aligned with traditional Chinese medicine (TCM), which aims to restore balance and slow cancer progression (Li J. et al., 2025; Li S. et al., 2025). Modern treatment strategies for genitourinary tumors are increasingly multimodal, incorporating immunotherapy (Ghatalia and Plimack, 2020), biological therapy, combination regimens, and even gut microbiota interventions (Fernandes et al., 2022). Similar to TCM, these approaches consider systemic balance to reduce cancer progression and complications. While targeted therapies have improved outcomes, drug resistance and side effects remain challenging. Therefore, multi-target, low-toxicity alternative and complementary and alternative medicine (CAM), including herbal remedies, are gaining attention.

In fact, complementary and alternative medicine (CAM) for urological tumors has gained widespread acceptance. For instance, phytotherapy within CAM is commonly used among patients with prostate cancer and bladder cancer, and these herbal therapies have also yielded positive outcomes in human randomized controlled trials (RCTs). This is because these medications offer the advantages of multi-targeted effects, multi-pathway mechanisms, and low toxicity (Kong et al., 2023). They can reverse drug resistance, synergize with conventional drugs, and target multiple aspects of tumor biology. However, complexity in composition, variability in processing, and differences in patient physiology make it difficult to achieve consistent biological concentrations, hampering clinical adoption and robust RCTs (Peng et al., 2022). Although particularly popular in Asian countries, the safety, efficacy, cost-effectiveness, and mechanisms of most CAM therapies remain poorly understood. The lack of high-quality clinical research to clearly establish the efficacy of different types of CAM interventions hinders the decision-making of healthcare practitioners or patients. These factors are largely attributable to the complexity of CAM drug components. Of course, this is also inextricably linked to variations in CAM drug dosages, differences in processing and formulation methods, as well as patients’ physiological conditions and adaptability (Peng et al., 2022).

**FIGURE 1:**
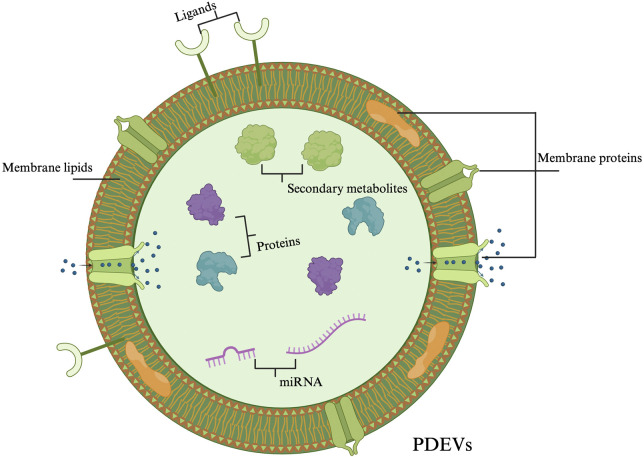
Schematic representation of key components commonly found in plant-derived extracellular vesicles (PDEVs). These include membrane lipids, ligands, secondary metabolites, proteins, microRNAs (miRNAs), and membrane proteins, which collectively contribute to the vesicles’ biological functions and potential applications.

Advances in nanomedicine and biotechnology have highlighted Plant-derived extracellular vesicles (PDEVs) as a new platform for cancer therapy (Figure 1). These natural nanoscale particles demonstrate excellent biocompatibility, anti-cancer activity, and tissue regenerative capacity, placing them at the cutting edge of innovative cancer strategies (Huang et al., 2023). Importantly, CAM plants-derived Extracellular vesicles (EVs) offer a more defined and quantifiable alternative to complex herbal mixtures, facilitating standardized evaluation. They carry mRNA, miRNA, bioactive lipids, and proteins, which can be analyzed through established methods (Zh et al., 2016). Moreover, their low biohazard risk, wide availability, low cost, and efficient drug-loading capacity make CAM plants-sourced EVs a strong candidate as complementary agents in treating genitourinary cancers (L et al., 2024; Zhao et al., 2023; Ou et al., 2021).

## Plant-based alternative therapies for genitourinary tumors

Economic and medical disparities in developing countries limit access to precision medicine (Yi et al., 2020), underscoring the need for more treatment options to reduce disease burden and improve quality of life. TCM, with its multi-target, multi-pathway, and low-toxicity profile, is widely accepted as an alternative cancer therapy. With a history spanning nearly 2000 years (Xiang et al., 2019), TCM is now integrated into China’s healthcare system. Numerous herbal formulas, extracts, and natural compounds have been found to inhibit genitourinary tumor development through various mechanisms (Wang X. et al., 2018). Besides suppressing growth, invasion, and metastasis, TCM can reverse drug resistance and synergize with conventional drugs. Ancient texts like Shennong Ben Cao Jing document many herbs with anti-tumor properties. Current research shows that combining TCM with immunotherapy enhances anti-tumor effects (Li J. et al., 2025). TCM contains bioactive compounds—such as alkaloids, polysaccharides, and flavonoids—that modulate immune function, affecting both innate and adaptive immunity (Table 1) (Xie et al., 2023).

**TABLE 1:** Effects of plants and their extracts on urogenital tumor cells.

Plant	Compound	Tumor Type	Mechanism	References
*Turmeric*	Curcumin	Prostate cancer	Inhibits prostate cancer proliferation by affecting multiple signaling pathways	Gururaj et al. (2002)
*Pomegranate*	Phenolic compounds punicalagin and ellagic acid	Prostate cancer	Inhibits proliferation of prostate cancer cells by affecting the NF-κB pathway	Rettig et al. (2008)
*Green tea*	Epigallocatechin gallate (EGCG)	Prostate cancer	Induces apoptosis and cell cycle arrest in prostate cancer cells	Masko and Freedland (2011)
*Green tea*	Epigallocatechin gallate (EGCG)	Bladder cancer	Inhibiting bladder tumor growth and invasion by modulating angiogenesis	Chen et al. (2004)
*Red Grapes*	Resveratrol	Prostate Cancer	Inhibits prostate cancer growth by regulating AR target gene expression through suppression of AR transcriptional activity	Shi et al. (2009)
*Milk Thistle*	Silybum marianum	Bladder cancer	Reducing recurrence of superficial bladder cancer through anti-angiogenesis	Raina et al. (2008)
*Green Tea*	Polyphenols	Bladder Cancer	Interference with the Carcinogenic Remodeling of Actin Components in Bladder Cancer Cells	Lu et al. (2005)
*Epimedium*	Flavone Glycoside II (ICS II)	Renal carcinoma	Inhibits proliferation, migration, and invasion of renal cell carcinoma cells via the ferroptosis pathway	Yu et al. (2022)
*Artemisinin*	Artemisinin Derivatives (ART)	Renal carcinoma	Inhibiting renal cell carcinoma growth by regulating cell cycle arrest and cyclin expression mechanisms	Markowitsch et al. (2020)
*Lycoris*	Lycorine	Renal carcinoma	Renal cell carcinoma Significantly inhibits the proliferation of renal cell carcinoma cells by regulating ferroptosis	Efferth (2017)
*Podocarpus*	Triacanthine	Bladder cancer	Inhibits proliferation, migration, and invasion of bladder malignancies by affecting matrix metalloproteinase (MMP)-9	Shin et al. (2019)
*Soybeans*	Soy isoflavones	Prostate Cancer	rostate cancer Influence on the proliferative activity of prostate cancer cells	Huang et al. (2024)
*Ganoderma lucidum*		Prostate Cancer	Inhibits cancer cell growth and survival while promoting apoptosis by blocking PI3K/Akt and MAPK/ERK signaling pathways	Sun and Sun (2019)
*Scutellaria baicalensis*		Prostate Cancer	Prostate cancer Inactivation of the PI3K/AKT signaling pathway induces apoptosis	Sheng et al. (2022)
*Snake Tongue Grass*		Prostate Cancer	Induces G2/M phase arrest in prostate cancer cells	Hnit et al. (2020)
*Coptis*	Aristolochic acid	Prostate Cancer	Prostate cancer Inhibits prostate cancer cell proliferation and promotes apoptosis	Sheng et al. (2022)
*Onion*	Quercetin	Prostate Cancer	Inhibits epithelial-mesenchymal transition (EMT) and promotes apoptosis	Hnit et al. (2020)
*Tripterygium wilfordii*	Triterpenoid alcohol	Prostate Cancer	Prostate cancer Inhibition of AR and its castration-resistant prostate cancer-associated variant AR-V7 phosphorylation via XPB/CDK7	Han et al. (2017)
*Astragalus*	Astragaloside IV	Prostate Cancer	Prostate cancer Inhibited the AKT/NF-κB signaling pathway, enhancing the sensitivity of prostate cancer cells to carboplatin	He et al. (2021)
*Patchouli*	Patchouli Alcohol	Prostate Cancer	Prostate Cancer Inhibits cancer cell growth and induces apoptosis through the NF-κB/Mcl-1 pathway	Cai et al. (2022)
*Corydalis*	Corydalin	Bladder cancer	Induces severe DNA damage via p53, leading to G2/M phase cell cycle arrest	Deng et al. (2023)
*Tiger Lily*	Irisin	Testicular Cancer	Regulating Proliferation and Expression of Stem Cell Factors to Inhibit Testicular Tumor Progression	Hasibeder et al. (2013)
*Olive Leaf*	Oleuropein	Testicular Cancer	Promotes Cancer Cell Apoptosis by Modulating Cell Cycle Regulators Through NF-κB Inhibition	Bossio et al. (2022)
*Alpinia oxyphylla Miq*	Nitazoxanide	Ovarian Cancer	Effectively promotes autophagy-mediated apoptosis in ovarian cancer cells via the Akt/mTOR signaling pathway	Lian et al. (2023)

### Renal cancer

Renal cancer is a disease with rising global incidence. As of 2022, there were 4,348,490 new cases and 155,953 deaths worldwide (Yu et al., 2024), and studies predict a continued increase in incidence over the next decade (Cirillo et al., 2024). Unlike early-stage localized renal cancer, which can be treated surgically, advanced and metastatic renal cancer is managed mainly with immunotherapy, targeted therapy, and chemotherapy (Chen et al., 2019). Renal cancer is a common and serious malignancy of the urinary system. While surgery is effective for early-stage renal cancer, advanced cases pose significant challenges due to poor treatment outcomes and chemoresistance (Yu et al., 2024). Hence, there is an urgent need to develop complementary and alternative treatments.

Recent studies have shown that ferroptosis is involved in the development and progression of renal cancer (Li et al., 2023; Zou et al., 2019; Hao et al., 2023). Ferroptosis-related genes can induce apoptosis and serve as potential biomarkers for early diagnosis, participating in chemoresistance in renal cancer. Icariside II (ICS II), a flavonoid with antitumor activity extracted from the traditional Chinese herb *Epimedium koreanum*, was found in a 2022 study to inhibit the proliferation, migration, and invasion of renal cell carcinoma (RCC) cells, an effect closely related to ferroptosis. Artesunate (ART), a derivative from the Chinese herb *Artemisia annua*, was studied in sunitinib-resistant RCC cell lines Caki-1, 78-O, KTCTL-26, and A-498 (Markowitsch et al., 2020), ART significantly inhibited tumor cell growth and survival by modulating ferroptosis-related molecular mechanisms. Overall, ART may offer a promising adjuvant treatment for advanced or drug-resistant renal cancer. Lycorine, a compound extracted from plants of the Amaryllidaceae family, is commonly used in TCM. It has various biological activities, including antiviral, antimalarial, anti-inflammatory, and antitumor effects, with relatively low side effects (Xiao et al., 2022). Studies indicate that lycorine significantly inhibits the proliferation of RCC cells, and its antitumor effect in renal cancer may be related to the induction of ferroptosis (Du et al., 2021). *Luteolin* (Lut), a natural flavonoid widely found in fruits and vegetables, has demonstrated potent anticancer activity (Chen et al., 2023). Lut significantly inhibits the survival of renal cancer cells *in vivo* and *in vitro*, accompanied by intracellular iron overload and abnormal depletion of glutathione (GSH) (Han et al., 2022).

In renal tumor immunotherapy, some Chinese herbs are believed to inhibit renal cancer progression through immunomodulation, enhancing antitumor immune responses. For example, polysaccharides in *Codonopsis* pilosula contribute to its antitumor properties through immunomodulatory effects (Liu et al., 2015) Astragalus membranaceus contains various polysaccharides, saponins, and other compounds that can modulate tumor immunity, affect tumor cell autophagy, and inhibit tumor angiogenesis (Guo et al., 2015). Interestingly, TCM is also thought to remodel immunosuppressive cells, such as reversing the immune phenotype of T lymphocytes and tumor-associated macrophages, promoting dendritic cell maturation, inhibiting myeloid-derived suppressor cell function, and regulating Th1/Th2 factors to modulate the tumor immunosuppressive microenvironment (Guo et al., 2015). In fact, active components in TCM exhibit complex and diverse mechanisms in tumor immunomodulation, such as promoting T-cell proliferation and dendritic cell (DC) maturation, inhibiting immune checkpoints and myeloid-derived suppressor cells (MDSCs), modulating the polarization of tumor-associated macrophages (TAMs), and enhancing the activity of T cells, natural killer cells (NKs), and TAMs (Xie et al., 2023).

Moreover, TCM is considered to have the advantage of multi-pathway, multi-target action in renal tumor therapy (Zheng et al., 2021), indicating broad-spectrum anti-renal cancer activity (Mo et al., 2025). For instance, curcumin and quercetin can target multiple pathways related to tumor proliferation, thereby increasing tumor cell sensitivity to chemotherapy and radiotherapy (Wang et al., 2023; Tang et al., 2020). Studies using the TCM systems pharmacology database and analysis platform have identified Chinese herbs involving multiple targets and signaling pathways related to renal tumorigenesis and glycolytic metabolism, potentially providing options for renal cancer immunotherapy.

In summary, plant-based alternative therapies may have potential value in the proliferation, metabolism, immunotherapy, and advanced drug resistance of renal cancer.

### Urothelial carcinoma

Urothelial bladder cancer is one of the most common malignancies of the urinary system, accounting for 90%–95% of urothelial carcinomas (Zhang et al., 2024). Most urothelial carcinomas are transitional cell carcinomas, including superficial or muscle-invasive lesions. Although the standard neoadjuvant treatment for high-grade muscle-invasive bladder cancer (T2-4 cN0 M0) is cisplatin combined with radical cystectomy, treatment outcomes remain suboptimal, with limited overall survival benefits. Although immunotherapy and immune checkpoint inhibitors have been established as first- or second-line treatments for localized and advanced urothelial cancer, improving overall survival in patients with high-grade muscle-invasive bladder cancer, the lack of robust evidence and the presence of adverse reactions (especially in elderly patients) mean that neoadjuvant immunotherapy is rarely used for such patients and is typically reserved for those with metastatic disease (Zhang et al., 2024). In fact, urothelial cancer cells proliferate and metastasize through multiple steps involving uncontrolled cell cycle regulation, signaling pathways, and apoptosis (Black and Dinney, 2007). Therefore, managing the molecular events associated with uncontrolled proliferation, migration, and invasion of urothelial cancer cells is a key target for developing new therapeutic drugs.

Recently, due to the potential efficacy and safety of current antitumor drugs, developing anticancer drugs from natural medicinal plants has attracted widespread attention. For example, *Cephalotaxus fortunei* can inhibit the proliferation, migration, and invasion of bladder cancer cells *in vitro* and in xenograft tumors. Further research found that cisplatin at a dose of 5 mg/kg caused significant weight loss, whereas an equivalent dose of triacanthine did not induce weight loss or behavioral changes (Shin et al., 2019). In terms of cell proliferation, triacanthine significantly and dose-dependently reduced bladder cancer cell proliferation, leading to accumulation of cells in the G1 phase (Shin et al., 2019). Ultimately, it was found that triacanthine effectively inhibited the growth and G1-S cell cycle transition of bladder cancer cells, induced apoptosis and autophagy, and effectively inhibited the binding of transcription factors such as AP-1, Sp-1, and NF-κB, ultimately suppressing the proliferation process of cancer cells (Shin et al., 2019). Studies on fruit extracts from Gleditsia sinensis also suggested inhibitory effects on urothelial carcinoma, significantly suppressing cancer cell proliferation and initiating the mitochondrial pathway of apoptosis (Cheung et al., 2005). Additionally, common vegetables may be potential alternative treatments for urothelial carcinoma. For instance, high intake of vegetables can reduce the risk of bladder cancer (Gl and ade, 1999). Subsequent meta-analyses have also shown that individuals with high dietary intake of fruits and vegetables have a reduced risk of bladder cancer (Steinmaus et al., 2000). Currently, a clinical trial on *green tea* polyphenol extract and EGFR inhibitors is recruiting patients to further determine the role of *green tea* in bladder cancer chemoprevention (Leppert et al., 2006). One factor is that *green tea* extract can interfere with the oncogenic remodeling of actin components in human bladder cancer cell lines (Lu et al., 2005). It can also inhibit bladder cancer development by modulating angiogenesis in the tumor microenvironment and arresting the cell cycle. Another factor is that *green tea* extract is a natural product metabolized by the kidneys and excreted, leading to long-term exposure of the urinary system to high drug concentrations, thereby achieving sufficient efficacy. Unfortunately, current evidence supporting the role of *green tea* in bladder cancer is mostly limited to *in vitro* studies. Some natural compounds have also been tested. Silibinin, a natural phenolic compound belonging to the flavonolignan family, is derived from the seeds of milk thistle (Raina et al., 2008). The chemopreventive and chemotherapeutic effects of silibinin in bladder cancer have been studied. Experiments assessed the effectiveness of intravesical silibinin in preventing the recurrence of superficial bladder tumors in rats, concluding that silibinin could be used via intravesical instillation to reduce the risk of recurrence in superficial bladder cancer, especially after transurethral resection (Zeng et al., 2011).

In summary, limited to animal models and cell experiments, natural plants show inhibitory effects on urothelial carcinoma, possibly through interfering with the cell cycle, tumor microenvironment, and other processes. Due to challenges in achieving therapeutic concentrations in patients, it is challenging to determine the chemotherapeutic potential of such drugs in genitourinary malignancies. Furthermore, while many natural drugs show promise in bladder cancer prevention and treatment, none can currently be recommended as a proven chemotherapeutic strategy. Identifying biological targets for alternative and complementary drugs may facilitate the application of natural plant drugs in urothelial carcinoma treatment.

### Prostate cancer

Prostate cancer (PCa) is one of the most common malignancies in men worldwide. According to 2023 statistics, new cases of PCa account for 29% of all new malignancies in men, and its mortality rate is second only to lung cancer, reaching 11% (Huang et al., 2024). This poses a significant threat to global male health. Common treatments for PCa include active surveillance, surgery, radiotherapy, conservative therapy, or androgen deprivation therapy (ADT) (Freedland et al., 2013). Precision medicine has provided more treatment options for prostate cancer patients, but issues of accessibility, drug resistance, and adverse reactions still need attention. Side effects associated with ADT increase the risk of non-cancer-related death (Nguyen et al., 2018), and AR-mediated resistance to abiraterone and enzalutamide remains a problem (Antonarakis et al., 2014). Imbalanced economic levels and medical conditions in developing countries also limit the application of precision therapy (Yi et al., 2020) Moreover, due to low PD-L1 expression and poor immunogenicity in prostate cancer, the new anticancer approaches PD-1 and PD-L1 inhibitors have not yet been applied in clinical treatment of prostate cancer (Rathi et al., 2021). Therefore, more treatment options are urgently needed to reduce the burden of this disease, especially for elderly men.

Currently, the use of natural extracts for treating PCa is increasing, with particular focus on specific foods rich in phytochemical compounds, such as *green tea*, tomatoes, soy isoflavones, curcumin, and glucosinolates from broccoli (Huang et al., 2024). Epidemiological studies have found that plant-based foods like cruciferous vegetables, garlic, tomatoes, pomegranates, and *green tea* are associated with a significant reduction in prostate cancer progression. As a representative of plant-based therapy, TCM has been widely accepted as an alternative cancer treatment due to its multi-target, multi-pathway, and low-toxicity advantages. An increasing number of herbal formulas, single extracts, and natural compounds have been discovered to inhibit prostate cancer development through various pathways (Wang X. et al., 2018). While suppressing the growth, invasion, and metastasis of prostate cancer, TCM can also reverse drug resistance and synergize with drugs. Chinese herbs show effects including inducing apoptosis and cell cycle arrest in prostate cancer cells, reversing drug resistance, and enhancing antitumor immunity. Mechanistically, they primarily affect signaling pathways such as PI3K/Akt/mTOR, AR, EGFR, and Wnt/β-catenin, which are often closely related to prostate cancer progression (Kong et al., 2023) As part of herbal formulas, single herb extracts exhibit various anti-prostate cancer cell activities, such as the anticancer bioactivity of *Ganoderma* lucidum and its extracts. In metastatic prostate cancer cells, ethanol extract of *Ganoderma* can block the PI3K/Akt and MAPK/ERK signaling pathways, which are related to cell growth, survival, and apoptosis (Huang et al., 2019). *Scutellaria barbata* (Banzhilian) from the Lamiaceae family has been shown *in vitro* and *in vivo* to induce apoptosis and G2/M cell cycle arrest by inactivating the PI3K/AKT signaling pathway, while also possessing anti-angiogenic properties (Sheng et al., 2022), effectively inhibiting the proliferation and metastasis of prostate cancer (Hnit et al., 2020).

However, due to the complexity of plant components, studies on whole plants are relatively scarce, making it difficult to elucidate the overall distribution of active components, which hinders comprehensive understanding through biochemical analysis. Extracting active components from Chinese herbs may help avoid this problem. Current development of new TCM drugs should focus more on component-based drugs and high-tech drug monomers (Zhang and Zhang, 2015), such as natural compounds extracted from plants like curcumin, aristolochic acid, quercetin, and triptolide. Curcumin can inhibit the proliferation and metastasis of PC-3 and DU145 cells and promote their apoptosis by inducing miR-30a-5p expression and inhibiting PCLAF (Pan et al., 2021). Curcumin can also induce ROS production, DNA damage, subsequent mitochondrial dysfunction, and promote apoptosis and necroptosis in PC-3 cells (Lee et al., 2021). Aristolochic acid promotes the expression of GADD45B, synergistically inhibiting prostate cancer cell proliferation and promoting apoptosis (Huang et al., 2018). It can also reduce the expression levels of MMP-2 and MMP-9 through inactivation of the NF-κB pathway, thereby inhibiting the migration and invasion of castration-resistant prostate cancer cells (Huang et al., 2017). Quercetin is a polyphenolic flavonoid widely found in Chinese herbs. It inhibits the epithelial-mesenchymal transition (EMT) process, downregulates MALAT1 expression in a dose- and time-dependent manner, promotes apoptosis, and inactivates the PI3K/Akt signaling pathway in the human prostate cancer cell line PC-3 (Lu et al., 2020). Quercetin modulates ROS production, interferes with MAPK, Akt, and NF-κB signaling pathways, and targets prostate cancer cells with different AR statuses (Lu et al., 2020). Triptolide is an active component of the Chinese herb *Tripterygium wilfordi*i. It inhibits the phosphorylation of AR and its castration-resistant variant AR-V7 at Ser515 via XPB/CDK7, enhancing the anti-prostate cancer activity of enzalutamide in castration-resistant prostate cancer (Han et al., 2017).

In summary, for PCa, therapeutic outcomes of various natural extracts may vary significantly, with both advantages and disadvantages. TCM extracts and natural compounds may provide alternative and complementary options for the treatment of advanced prostate cancer (CRPC) due to their roles in inducing apoptosis, autophagy, interfering with the cell cycle, inhibiting angiogenesis, proliferation, and migration, reversing drug resistance, and enhancing antitumor immunity. A retrospective cohort study involving 1132 prostate cancer patients showed that longer use of TCM was associated with a lower risk of death (Liu et al., 2016). However, there is a significant gap between experimental data and clinical application. In basic experiments, curcumin exhibits various anti-prostate cancer cell activities, but it did not show corresponding therapeutic effects in randomized controlled studies (Pas et al., 2021). This may be because the human body is a whole, and different microenvironments may affect cancer cell responses to Chinese herbs. *In vitro* cell lines are in a relatively stable state, whereas *in vivo* tumor cells are in a more active state, which is difficult to simulate *in vitro* experiments.

### Germ cell tumors

Germ cell tumors (GCTs) are tumors arising from germ cells. They can be malignant or benign and typically occur in the gonads (ovaries or testes), primarily due to congenital defects during embryonic development. Testicular germ cell tumors (TGCTs) are rare cancers in children, adolescents, and young adults, with incidence continuously rising over the past four decades (Shen et al., 2016). TGCTs have diverse biological characteristics, originating from a precursor lesion called germ cell neoplasia *in situ*, which can grow *in situ* within the seminiferous tubules and express transcription factors shared with embryonic stem cells. Despite a common cell of origin, testicular cancers are histologically and clinically classified into seminomas and non-seminomas, including embryonal carcinoma, yolk sac tumor, choriocarcinoma, and teratoma (Hayes-Lattin and Nichols, 2009).

Research has found that certain traditional Chinese medicines exert an influence on the progression of germ cell tumors. The rhizome of *Belamcanda chinensis* is well-known in TCM for treating various symptoms and diseases. To date, multiple compounds have been identified from its extracts, including several phytoestrogens (Wagner et al., 2011). The anticancer effects of phytoestrogens have been demonstrated in various cancers and cell lines. On one hand, the epidemiological incidence of malignancies is thought to be related to the abundance of (phyto)estrogens (Adlercreutz, 1995). On the other hand, their popularity in the population makes them candidates for potential drugs or adjuvant therapies. For example, postmenopausal women prefer taking phytoestrogens over traditional hormone replacement therapy, considering the latter “unnatural” (Glazier and Bowman, 2001). Although germ cell tumors (TGCT) are relatively rare and highly curable, affected young patients face long-term adverse consequences from chemotherapy toxicity or radiotherapy side effects, which may negatively impact fertility and sexual activity (La Vignera et al., 2019; Paoli et al., 2018). Recently, a growing body of literature reports that plant extracts may play a role in developing new anti-testicular cancer drugs. Studies found that oleuropein (OLE), the main polyphenol component in olive leaf extract, reduces testicular tumor cell growth by promoting apoptosis and counteracting cell motility and migration (Bossio et al., 2022).

Ovarian cancer (OC) is the eighth most common cancer in women globally and a major gynecological disease with high mortality (Biller et al., 2021). The pathogenic mechanism of ovarian cancer is not fully understood and is generally considered a result of genetic and environmental interactions. Cytoreductive surgery is an effective treatment for ovarian cancer, but its efficacy remains limited for advanced patients. Currently, platinum-based combination chemotherapy is the first-line treatment for advanced ovarian cancer (Zhang et al., 2021). However, 20% of patients do not respond to platinum chemotherapy initially, and up to 75% of ovarian cancer patients experience recurrence. Meanwhile, patients treated after recurrence often develop resistance to chemotherapy drugs (Friedlander et al., 2018). Some TCM components have been found to have anticancer effects, inhibiting cancer cell proliferation and inducing apoptosis, thereby enhancing patient sensitivity to chemotherapy drugs *in vivo* and/or *in vitro*, such as daidzein, curcumin, phytosterols, ginsenosides, quercetin, etc., (Atanasov et al., 2021; Xu et al., 2022). Their mechanisms of action include affecting gene expression, antioxidant effects, inhibiting the activity of key enzymes in cell metabolism, and inhibiting tumor angiogenesis (Ge et al., 2021). Active components of TCM, as adjuvants to chemotherapy, have broad clinical application prospects. Alkaloids extracted from the roots or stems of Alpinia oxyphylla can induce apoptosis and autophagy in ovarian cancer cells, suggesting they may help improve the sensitivity of ovarian cancer cells to chemotherapy drugs (Lian et al., 2023).

In summary, TCM can serve as a long-term complementary and alternative therapy because it shows benefits in alleviating clinical symptoms of germ cell tumors, reducing the toxic side effects of radiotherapy and chemotherapy, and improving patient quality of life. However, due to the rarity of germ cell tumors, research on complementary and alternative treatments for these tumors is still very limited. Due to differences in species and the absorption and transformation of TCM *in vivo*, multi-species and large-scale studies are needed for confirmation.

## Potential mechanisms of plant-derived extracellular vesicles in genitourinary tumors

**FIGURE 2:**
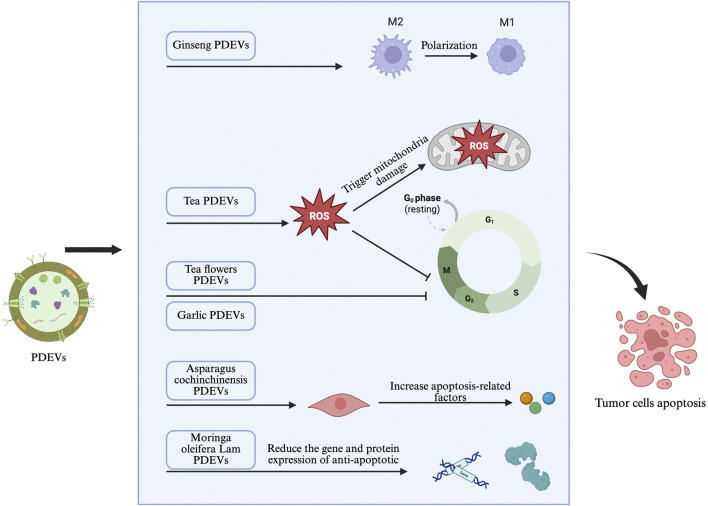
Potential mechanisms of PDEVs in genitourinary tumors.

Advances in nanobiotechnology have revealed that PDEVs represent a novel and promising approach for cancer therapy. Due to their unique anticancer properties, biocompatibility, and targeted delivery capabilities, they are at the forefront of innovative cancer treatment strategies (Huang et al., 2023). Their main anticancer characteristics give them great potential in new paradigms of cancer treatment (Figure 2). These anticancer properties primarily include inducing cell cycle arrest, promoting apoptosis pathways within tumor cells, inhibiting cell metastasis, activating antitumor immunity, and altering the tumor microenvironment in various types of cancer. Of course, the composition and bioactivity of PDEVs are major factors influencing anticancer properties, depending on the donor cells, i.e., specific parts of the plant, indicating the diversity and complexity of PDEV anticancer functions. As such, PDEVs can act on various tumors, including genitourinary tumors, through different functions and therapeutic purposes (Kim et al., 2023). These common tumor-suppressive mechanisms of PDEVs may be potential mechanisms for their application in genitourinary tumors, so understanding and summarizing these pathways will aid in the development of PDEV-based treatment systems for genitourinary tumors.

### Interference with cell proliferation

Many natural plants exhibit cytotoxic effects, mainly due to interference with the cell proliferation cycle, and their derived extracellular vesicles show similar effects. EVs extracted from two plants known for cytotoxicity—*Acanthopanax senticosus*(At a concentration of 50 μg/mL, the inhibition rate of malignant tumor cells exceeded 60%.) and *Pinus densiflora*(At a concentration of 50 μg/mL, the inhibition rate of malignant tumor cells was less than 40%.)—exhibited strong cytotoxicity against tumor cells with synergistic effects. Further research found that this cytotoxicity primarily affected cancer cell cycle proteins, thereby interfering with cell proliferation. In contrast, EVs extracted from other known cytotoxic plants (*Abies balsamea* and *Chamaecyparis obtuse:* At a concentration of 50 μg/mL, the viability of malignant tumor cells remained above 70%) had no significant effect on any tumor cell type (Kim et al., 2020). This indicates that PDEVs from specific sources (not all EVs from known anticancer plants) represent an effective and selective nanomedicine for malignant tumor cells. Interestingly, these PDEVs are cytotoxic to tumor cells but not to normal cells (Kim et al., 2020). This selective cytotoxicity suggests that PDEVs may have fewer side effects compared to traditional chemotherapy drugs and could synergize with conventional anticancer drugs. It is suggested that tumor cells and normal cells use different endocytic pathways to uptake PDEVs, and the caveolin-mediated endocytic pathway may prevent their degradation and allow accumulation of PDEVs, which could be the main reason for cellular selection differences (Kim et al., 2020). Of course, further research on the endo-lysosomal pathway mechanism is needed. Due to the cancer-preventive effects of foods, the anticancer effects of fruit-derived PDEVs have also attracted attention. Studies show that some fruit-derived PDEVs have significant anti-proliferative effects, giving them anticancer properties. For example, *lemon*-derived EVs have been shown to significantly inhibit tumor cell proliferation (Raimondo et al., 2015). Bioactive PDEVs isolated from *lemon* juice exert anticancer cell proliferation effects *in vitro* and *in vivo*. This antitumor mechanism is confirmed to be related to PDEV-induced ROS production, which upregulates GADD45a, leading to arrest in the S phase of cancer cell proliferation. In fact, even within the same genus, plants can have different anticancer mechanisms. Scholars analyzed PDEVs from four citrus species (*C. sinensis*, C. limon, C. paradisi, and C. aurantium) and found that all four could inhibit the growth of A375 (human melanoma), A549 (human lung cancer), and MCF-7 (human breast cancer) cells. However, only vesicles from C. paradisi could block the G2/M phase of tumor cells by enhancing gene expression of CDKN1A (encoding p21) and reducing levels of cyclin B1 and cyclin B2 (Stanly et al., 2020). Besides differences in plant species, PDEVs produced from different parts of the same plant can also have varying anticancer effects. For example, vesicles from the roots of Dendropanax morbifera inhibited tumor proliferation by downregulating the expression of TYR, TRP-1, and TRP-2 genes, but vesicles from its leaves had a stronger effect (Cao et al., 2019). The anticancer activity of some plant compounds is also reflected in their extracted PDEVs. For instance, plant compounds like curcumin and quercetin are present in their extracted PDEVs, which can interfere with the cancer cell proliferation cycle by regulating Nrf2, MAPK, and NF-κB signaling pathways, thereby inhibiting cancer cell spread (Liu et al., 2021). The content of intrinsic anticancer active drugs in PDEVs also determines their inhibitory effect on the cancer cell proliferation process. Studies show that PDEVs from Cannabis sp. can be divided into high-cannabidiol (CBD) and low-CBD categories. Only high-CBD PDEVs inhibited the cell proliferation of hepatocellular carcinoma cell lines HepG2 and Huh-7 in a time- and dose-dependent manner. This is because high-CBD vesicles arrested the cell cycle of liver cancer cells in the G0/G1 phase (Tajik et al., 2022). In fact, PDEVs extracted from various edible plants can also participate in inhibiting cancer cell proliferation through multiple pathways. It was found that in a colorectal cancer (CRC) mouse model, daily oral administration of 0.3 mg of *ginger*-derived PDEVs significantly reduced the mRNA expression of cyclin D1, thereby affecting rectal cancer progression. Notably, most traditional anticancer drugs are toxic to both malignant and normal cells, which can have serious consequences for healthy body tissues (Susanti et al., 2012). Hence, there is clinical advocate for new anticancer drugs with reduced side effects (Desai et al., 2008). Drugs developed based on PDEVs may combine anticancer effects with low adverse reactions. First, PDEVs have properties similar to mammals, enabling efficient transfer of plant components into mammalian cells to exert therapeutic effects (Zh et al., 2016). Second, their specific cell targeting may greatly reduce harmful reactions to healthy body tissues compared to raw plant compounds. For example, studying the anti-cell proliferation activity of garlic-derived PDEVs on two cancer cell lines (A498, renal cancer cells; A549, lung cancer cells) and one normal cell line (HDF), it was found that PDEVs specifically targeted cancer cells and accumulated intracellularly, significantly reducing cancer cell viability through S-phase cell cycle arrest, while accumulating minimally in normal cells and not causing cytotoxic effects on the normal cell line. This high selectivity difference may be due to differences in the composition of cancer cell membranes and normal cell membranes affecting cell uptake pathways (Stanly et al., 2020). This anti-proliferation effect specific to cancer cells can minimize side effects caused by non-specific interactions with normal tissues and improve the safety of treatment regimens (Vyas et al., 2014).

### Promotion of apoptosis

In addition to affecting cell proliferation, PDEVs can also control cancer development by promoting apoptosis (Chintapula et al., 2024). In fact, many naturally derived compounds show unique anticancer properties by inducing apoptotic pathways in tumor cells, such as *bitter melon* extract (Ru et al., 2011; Kohno et al., 2002). However, single compounds often have strong cytotoxicity, leading to severe side effects. Increasing studies show that plant-derived PDEVs have potential anticancer effects in various cancer models, primarily by interfering with the apoptosis process of cancer cells. For example, *lemon*-derived PDEVs can induce TNF-related apoptosis-inducing ligand (TRAIL)-mediated apoptosis through the TRAIL/Dr5 pathway without affecting normal cell survival (Ishiba et al., 2008; Koornstra et al., 2003; Yagita et al., 2004). PDEVs isolated from *turmeric* were found to exhibit apoptotic effects on cancer cells *in vitro* without adverse effects on normal colon epithelial cells (Sasaki et al., 2023). Therefore, the anticancer benefits of PDEVs from specific sources may be greater than those of the plant itself or its extracted compounds. Based on active substances derived from certain plants, such as microRNA (miRNA) and small interfering RNA (siRNA), which have been shown to silence oncogenes or activate tumor suppressor genes, and specific proteins and key peptides that can induce cancer cell apoptosis, these biomolecules can be carried by PDEVs and targeted delivered to cancer cells (Huang et al., 2023). For example, overexpression of NLRP3 is found in oral squamous cell carcinoma, leading to resistance to 5-fluorouracil (5-FU) (Feng et al., 2017; Wang H. et al., 2018). The combination of *bitter melon*-derived PDEVs and 5-FU improved the therapeutic effect on oral squamous cell carcinoma (Yang et al., 2021). Compared to treatment with 5-FU or *bitter melon*-derived PDEVs alone, tumor cells receiving combination therapy showed a significant increase in apoptosis. Specifically, bitter melon-derived PDEVs activated the MEK-ERK and p38MAPK pathways, thereby inducing apoptosis (Yang et al., 2021). Besides common dietary plants, some Chinese medicinal plant-derived PDEVs have similar effects. For example, PDEVs extracted from *Dendrobium* could inhibit tumor cell growth by inducing cancer cell apoptosis, mainly because PDEVs targeted and increased the levels of apoptosis-related factors (such as AIF, Bax, and Bak) in various human hepatocellular carcinoma cells (Hao et al., 2024). Cannabis-derived PDEVs have also been shown to induce cell death in hepatocellular carcinoma by triggering mitochondrial-dependent apoptotic signaling pathways (Tajik et al., 2022). In summary, various plant-derived PDEVs can inhibit cancer cell growth through the apoptosis process while weakening or even not affecting the survival of normal cells.

### Inhibition of cell metastasis

Currently, cancer discovered with multiple metastases often represents advanced stages, with sharply declining survival, especially in the most common lung metastases. Inhibiting cancer cell metastasis is considered an important part of anticancer drug exploration. Studies have found that besides inducing cell cycle arrest and promoting tumor cell apoptosis, PDEVs also exhibit anti-tumor metastasis properties. Considering that most cancers tend to metastasize to the lungs, researchers have focused on the therapeutic effects of PDEVs on lung metastasis. Studies found that certain PDEVs can inhibit cancer metastasis to the lungs *in vivo* through regulation of reactive oxygen species (ROS) production and microbiota (Chen Q. et al., 2022). For example, *lemon* is known to contain polymethoxyflavones (PMFs), bioactive compounds found only in citrus plants, which exhibit anti-lung metastasis properties in various cancer models (Wang L. et al., 2014). Their derived PDEVs also contain PMFs, and certain concentrations of PDEVs showed strong anticancer and anti-lung metastasis capabilities against colon cancer cell lines *in vitro* and *in vivo* (Sasaki et al., 2023; Sasaki et al., 2021). Besides active anticancer compounds, PDEVs can also effectively deliver anticancer miRNA molecules to tumor sites. For example, *grapefruit*-derived PDEVs were shown to deliver miR-18a to liver malignancies and inhibit their distant metastasis (Wang et al., 2015). This is because miR-18a-containing PDEVs promoted increased activity of M1 macrophages, enhancing secretion of IFNγ and IL-12 and inhibiting release of TGFβ and IL-10 by M2 cells, thereby stimulating and recruiting natural killer cells and T cells to the tumor site and reducing distant metastasis of tumor cells (Wang et al., 2015). Similarly, most plant-derived PDEVs control distant cancer metastasis by affecting the immune status *in vivo*. For example, the NLRP3 inflammasome is a key component of the innate immune system and plays an important role in cancer spread and growth (Moossavi et al., 2018; Ju et al., 2021). In oral squamous cell carcinoma, NLRP3 can accelerate tumor growth and metastasis, and many factors including K+ efflux, intracellular calcium, endoplasmic reticulum (ER) stress, and ROS can activate the NLRP3 inflammasome (Wang H. et al., 2018), including *bitter melon*-derived PDEVs. These PDEVs could inhibit distant metastasis of oral squamous cell carcinoma by downregulating NLRP3 expression (Wang H. et al., 2018). Previously, plant-derived PDEVs were thought to be mainly used for regulating inflammation, tissue repair, and gut microbiota. In fact, regulating these pathways can further affect cancer cell spread and metastasis (Xu et al., 2023; Yang et al., 2023).

### Activation of immune response

Controlling cancer progression and recurrence through immunomodulatory pathways is gaining increasing attention, and plant-derived PDEVs have been confirmed to inhibit tumor growth by activating immune processes. It has been found that some plant-derived PDEVs participate in intercellular communication in plants as a means of regulating plant innate immunity (Rutter and Innes, 2017). Thus, it can be hypothesized that PDEVs from specific plants may also act as immune enhancers to inhibit tumor progression. This is indeed the case. *Grapefruit*-derived PDEVs can enhance host immune responses to inhibit tumor cell progression by improving immunomodulatory effects (Wang B. et al., 2014). These PDEVs exhibited dose-dependent cytotoxic effects *in vitro*, and their anticancer effects lasted for a relatively long time (Wang B. et al., 2014). Mechanistically, these PDEVs can alter the polarization of M2 macrophages to enhance anti-tumor immune responses (Cao et al., 2019). Specifically, this immune-enhancing effect is mediated by macrophages, shifting M2-like macrophages from an immune-supportive (CD11b+ F4/80+ CD206+) phenotype to a more tumoricidal M1-like (CD11b+ F4/80+ CD86^+^) state. Sustained M1 polarization maintains cytokine production and cytotoxic hydroxyl radical generation, thereby inducing cancer cell apoptosis (Cao et al., 2019). In the tumor microenvironment, mouse macrophages can express TLR1-9, TLR11, and TLR13, which play roles in macrophage activation, differentiation, and polarization (Cao et al., 2019). PDEVs can trigger macrophage polarization in a TLR4/MyD88-dependent manner (Cao et al., 2019). Besides affecting macrophage polarization, PDEVs can also induce activation of the adaptive immune system. Pro-inflammatory cytokines produced by PDEV-activated macrophages activate Th1 immune responses, promoting the activation of CD8^+^ T cells, leading to targeted killing of tumor cells (De Henau et al., 2016; Wammers et al., 2018). For example, PDEVs from *Petasites japonicus* can act as immune adjuvants to activate immune responses. After treating dendritic cells, they strongly induced the proliferation and differentiation of naive T cells into Th1-type T cells and cytotoxic CD8^+^ T cells, increasing the secretion of interferon-γ and interleukin-2 (Han et al., 2021). Catharanthus roseus-derived PDEVs could also increase macrophage immune activity and polarization through activation of the TNF-α/NF-κB/PU.1 process. Thus, we can hypothesize that PDEVs can not only directly inhibit tumor cell progression but also be used as immune stimulants after chemotherapy (Ou et al., 2023).

### Alteration of tumor microenvironment

The tumor microenvironment (TME) is markedly different from the normal physiological environment and is a prerequisite for supporting tumor cell proliferation and metastasis. An inflammatory microenvironment significantly promotes cancer proliferation, metastasis, and drug resistance. A relatively hypoxic environment promotes angiogenesis, providing sustained nutrient supply for cancer cell growth. Tumor-associated macrophages (TAMs) are a major component of the TME (Noy and Pollard, 2014). Studies show that TAM infiltration in tumor tissue supports tumor growth, angiogenesis, invasion, and metastasis, and is positively correlated with tumor progression and drug resistance (Mantovani et al., 2002). Generally, TAMs have considerable plasticity and exhibit opposite phenotypes and functions, including tumor-killing M1 macrophages and tumor-supporting M2 macrophages. In most tumor types, M2-like phenotype macrophages dominate. Clearing M2-like macrophages and tilting macrophages towards the M1-like phenotype has become an attractive strategy in cancer therapy (Biswas and Mantovani, 2010). In fact, PDEVs can inhibit cancer progression through the above processes, emphasizing their ability to manipulate the cellular microenvironment towards a state that suppresses tumor cell growth (NCT01294072 and NCT01668849). Studies found that *ginseng*-derived PDEVs could inhibit M2-like polarization of macrophages and promote M1 macrophage expression in the TME through a mechanism dependent on the Toll-like receptor (TLR)-4/myeloid differentiation primary response 88 (MyD88) signaling pathway. *In vivo* experiments showed significant inhibition of certain tumor growth in mice treated with *ginseng*-derived PDEVs (Cao et al., 2019). Besides *ginseng*, PDEVs extracted from *carrots, apples, broccoli, ginger, grapes, grapefruit,* and *cabbage* have also been developed and evaluated for their anticancer properties (Raimondo et al., 2015; Mu et al., 2014). It is reported that these PDEVs can reduce cancer growth by modulating macrophage polarization and TAMs (Noy and Pollard, 2014; Biswas and Mantovani, 2010). Over the past decade, immune checkpoint inhibitors have gained attention in cancer therapy for their ability to promote cytotoxic T cell activity to kill cancer cells. However, due to insufficient T cell infiltration, immune checkpoint inhibitors are ineffective for some solid tumors, which are called cold tumors. Interestingly, some plant-derived PDEVs can enhance the anticancer effects of immune checkpoint antibodies by reprogramming the cold tumor microenvironment. This ability to “heat up” the “cold” tumor environment by reprogramming TAMs has prompted attempts to synergize PDEVs with immune checkpoint inhibitors, opening a new avenue for the treatment of solid tumors (Zhao et al., 2023). Additionally, due to active cancer cell metabolism, they are usually more susceptible to oxidative stress than normal cells, and increased ROS content in the cellular microenvironment can indirectly elevate intracellular ROS levels, triggering mitochondrial damage and blocking the cell cycle, thereby inhibiting cancer cell proliferation and migration. Inducing a large amount of ROS in the tumor environment can also induce cancer cell death, while most normal cells are spared. It is reported that edible tea flower-derived PDEVs can inhibit cancer development by producing excessive ROS with basically no effect on normal cells (Zhao et al., 2023). Moreover, high ROS can also increase the susceptibility of drug-resistant cancer cells. For example, *bitter melon*-derived PDEVs increased the chemosensitivity of squamous cell carcinoma cells to 5-fluorouracil (Zhao et al., 2023). In summary, most PDEVs can interfere with tumor progression by affecting the TME of cancer cells. However, certain TMEs also pose challenges for the application of PDEVs. For example, an acidic TME may cause premature degradation of PDEVs, and a high-immunity-status TME may interfere with PDEV surface proteins, preventing targeted fusion with tumor cells. Developing nanomedicine-modified delivery systems and strengthening the surface resistance of PDEVs may help solve these problems.

## Plant-derived extracellular vesicles as potential adjuvants for anti-genitourinary tumor therapy

**FIGURE 3:**
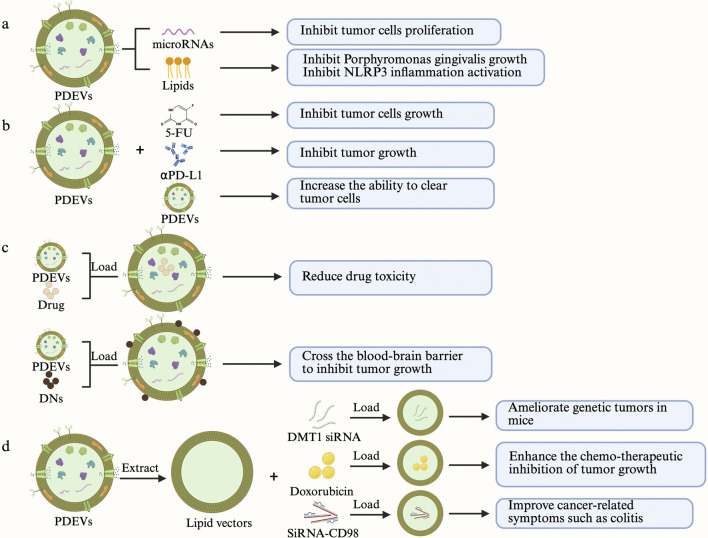
PDEVs as potential adjuvants for anti-genitourinary tumor therapy. **(a)** PDEVs serve as drugs to treat tumors. **(b)** PDEVs are synergistically used with other drugs to treat tumors. **(c)** Isolated PDEVs are directly loaded with drugs to treat tumors. **(d)** Extracted and reassembled PDEVs are loaded with drugs to treat tumors.

Traditional chemotherapy drugs kill rapidly proliferating cancer cells by obstructing the cell proliferation cycle (Bertrand and Leroux, 2012). This low selectivity means that rapidly proliferating healthy cells are also adversely affected, which may cause or accelerate the death of cancer patients (Maas et al., 2017). On one hand, metastatic cancers often require high drug doses, which inevitably increases cytotoxicity to normal tissues (Skotland et al., 2017); on the other hand, subtherapeutic doses allow cancer cells to adapt to the microenvironment and acquire multidrug resistance (French et al., 2017). Therefore, complementary drugs that enhance the anticancer properties of chemotherapy drugs and reduce side effects are gradually being considered. In recent years, some TCM components have been found to inhibit cancer cell proliferation and induce apoptosis, improving patient sensitivity to chemotherapy drugs (Lian et al., 2023). Since the content of PDEVs depends on the cell type that releases them (Pegtel and Gould, 2019), most plant-derived PDEVs may also exhibit synergistic effects with chemotherapy and endocrine therapy (Figure 3). For example, plant-derived PDEVs can reduce PSA levels, delay the onset of castration-resistant prostate cancer, reduce adverse reactions to endocrine therapy, and prolong patient survival throughout the treatment process (Kong et al., 2023).

### Enhancement of antitumor effects

It is widely recognized that traditional Chinese herbal medicine can mitigate the toxic side effects of chemotherapy and radiotherapy, enhance the antitumor efficacy of these treatments, alleviate clinical symptoms caused by tumors, and ultimately prolong survival in postoperative and advanced cancer patients (Li et al., 2012). Similarly, their derived PDEVs have similar effects. *Black pepper*-derived PDEVs can enhance the ability of chemotherapy drugs to induce cancer cell apoptosis (Zhou et al., 2015). Plant-derived PDEVs loaded with chemotherapy drugs can improve the solubility and bioavailability of the drugs. The large surface area, strong targeting ability, and slow degradation characteristics of PDEVs reduce off-target effects, allowing effective sustained release within cancer cells. In fact, anticancer drugs loaded into PDEVs may also synergize with the intrinsic active substances in PDEVs (Qiang et al., 2024). For example, *Litsea cubeba*-derived PDEVs can transfer active miRNA to tumor cells, synergizing with chemotherapy drugs to inhibit angiogenesis and tumor development (Qiang et al., 2024). *Grapefruit*-derived PDEVs contain anticancer genetic material (Kameli et al., 2021). By synergistically loading the common chemotherapy drug doxorubicin to target tumor cells, they significantly enhanced its anticancer effect (Kameli et al., 2021). It can be assumed that common bladder cancer chemotherapy drugs can also be loaded by PDEVs to enhance synergistic anticancer effects. Moreover, for drug loading, engineering modifications further improve the targeting and drug loading efficiency of PDEVs. Using *ginger*-derived PDEVs, a targeted delivery platform was designed by modifying its folate ligand and loading doxorubicin. Compared to doxorubicin alone, it enhanced anticancer effects and greatly reduced the cardiotoxicity of doxorubicin (Zhang et al., 2016a). Additionally, through genetic engineering, genetically modified PDEVs also show synergistic anticancer and targeting potential, such as loading anticancer RNA, nucleic acids, etc. More importantly, medicinal plants are seen as valuable resources for screening and preparing PDEVs based on their unique anticancer properties, containing phytochemicals, proteins, and nucleic acids, offering cheaper and more convenient treatment options compared to synthetic drugs and animal protein-based drugs (Qiang et al., 2024).

### Reduction of drug resistance

Cancer cells often develop resistance to these drugs over time, reducing their therapeutic effectiveness and complicating treatment efforts. Although chemotherapy can temporarily reduce or eliminate tumor cells, recurrence remains a significant issue, and recurrent cancers often exhibit stronger drug resistance. For example, the high recurrence rate of urothelial carcinoma often indicates resistance to existing local chemotherapy drugs (Knowles et al., 2021). Unlike conventional treatments, which basically rely on pure compounds extracted from natural sources or artificially manufactured products that may develop resistance with long-term use, drug delivery based on PDEVs bypasses these problems (Di Gioia et al., 2020). Generally, PDEVs have no toxic effects on healthy cells but act on abnormal, transformed cancer cells (Zhang et al., 2016b). The factors by which PDEVs reduce chemotherapy drug resistance may include: first, the phospholipid bilayer can protect its cargo, ensuring therapeutic drugs remain intact until targeted delivery to the intended site (Zeng et al., 2023); second, modified PDEVs can support personalized treatment methods tailored to the patient’s genetics and tumor specifics (Rezaie et al., 2022). Traditional monotherapy faces considerable challenges, especially in addressing cancer multidrug resistance. To overcome multidrug resistance issues, focus has been placed on using multidrug resistance inhibitors (MDR inhibitors) or new anticancer drugs (Maimaitijiang et al., 2024). Using engineered EVs to co-deliver MDR inhibitors and chemotherapy drugs can effectively inhibit MDR and improve chemotherapy efficacy, which is considered a promising strategy. However, using animal-derived EVs, especially those from cancer cells, carries potential risks of promoting cancer and inducing anticancer drug resistance. For example, animal-derived EVs can activate fibroblasts, thereby enhancing signaling at metastasis sites and remodeling the distant tumor microenvironment to promote cancer cell metastasis (Webber et al., 2010). But plant-derived EVs do not show similar risks (Song et al., 2025). For example, *bitter melon*-derived PDEVs can synergize with 5-FU to enhance cytotoxic effects on tumor cells and reduce their resistance (Yang et al., 2021). Cancer is usually determined by a multi-point regulatory network, and existing drugs targeting single targets are difficult to achieve long-term efficacy, which may lead to drug resistance or severe side effects. Therefore, PDEV-based combination complementary therapies aimed at achieving multi-target synergistic treatment may be a potentially efficient anticancer strategy for genitourinary tumors.

### Reduction of adverse reactions

Many kinase inhibitor drugs attempt to avoid the toxic side effects of chemotherapy by targeting molecules unique to cancer cells (Jayasinghe et al., 2021). Although these drugs provide a more active way to target tumor cells, they also bring associated toxicities. PDEVs obtained from *grapefruit*, which contains large amounts of potent antioxidants, were found to contain measurable levels of ascorbic acid, catalase, and glutathione, which may help alleviate damage to normal liver cells caused by chemotherapy drugs (Castelli et al., 2023). Some plant compounds can also be used to improve side effects after cancer chemotherapy due to their antioxidant, anti-inflammatory, and immunomodulatory effects (Huang et al., 2023). However, single compounds may also cause serious side effects, such as nausea, vomiting, diarrhea, rash, hand-foot syndrome, etc., which significantly affect patient compliance and quality of life (Ghali et al., 2019). PDEVs extracted from Chinese herbal plants may help avoid these effects. For example, mice intravenously injected with PDEVs(Grapefruit-derived extracellular vesicles) containing folate (FA)-paclitaxel (PTX) showed significant tumor growth inhibition compared to mice receiving only PDEVs. At the same time, it was found that the modified PDEVs did not cause any adverse reactions or pathological changes in major organs such as lungs, kidneys, liver, or spleen (Wang et al., 2015). Modified *ginger* PDEVs were not only proven to successfully deliver cargo to target cancer cells without damaging major organs but also seemed to alleviate side effects usually caused by chemotherapeutic agents (Zhang et al., 2016b). In solid tumors, PDEVs can exhibit enhanced permeability and retention effects, achieving targeted accumulation in cancer cells. The phospholipid bilayer membrane structure of PDEVs enables them to fuse directly with cell membranes, thereby circumventing drug release and cytotoxicity issues associated with the phagolysosomal pathway (Zhang et al., 2022). These properties make PDEVs an ideal delivery system to reduce the adverse reactions of chemotherapy drugs. This remarkable specificity toward tumor cells and the reduced drug degradation mediated by endocytic pathways in mammalian cells indicate that the modified drug-loaded PDEVs exhibit lower toxicity to non-targeted cells and organs (Munir et al., 2020). In fact, one of the biggest side effects of chemotherapy drugs is that they stimulate the body’s immune response, severely damaging the function of vital organs through the release of large amounts of inflammatory factors (Adrian et al., 1980). PDEVs isolated from *cabbage* (Cabex) and *red cabbage* (Rabex) can effectively promote the proliferation of RAW 264.7 cells to suppress the degree of inflammation, thereby protecting cells from inflammatory stress damage (You et al., 2021). PDEVs from different plant sources have been tested for their anti-inflammatory effects in various models activating immune cells. In one such study, it was reported that *lemon*-derived PDEVs could inhibit macrophage activation via NF-κB and extracellular signal-regulated kinase (ERK) without changing the phenotype (Raimondo et al., 2022). Celery root-derived PDEVs had a significant inhibitory effect on the activation of T lymphocytes and peripheral blood mononuclear cells activated by chemotherapy drugs (Taşlı, 2022). PDEVs isolated from *papaya* could inhibit the polarization of neutrophils and macrophages, reduce levels of inflammatory cytokines (such as IL-1β and IL-6), and increase levels of anti-inflammatory cytokines (such as IL-10) (Iriawati et al., 2024). In 2012, researchers at the University of Louisville initiated a clinical trial (NCT01668849) to study the anti-inflammatory activity of grape-derived PDEVs to reduce the incidence of oral mucositis in tumor patients during radiotherapy and chemotherapy. Interestingly, PDEVs may also play an important role in maintaining gut microbiota symbiosis and intestinal homeostasis (Rufino-Ramos et al., 2017), which may help reduce intestinal adverse reactions to chemotherapy drugs, such as diarrhea and constipation. Grape-derived PDEVs can be taken up by intestinal stem cells, where they stimulate stem cell proliferation and self-renewal, suggesting the possibility of repairing chemotherapy-induced damage to intestinal function by consuming grape-derived PDEVs (Ju et al., 2013).

## Potential administration routes of plant-derived extracellular vesicles in genitourinary tumors

Although many breakthrough advances have been made in the treatment of genitourinary malignancies over the past decade, there is still significant room for improvement (Kathuria-Prakash et al., 2024). Excellent administration routes for genitourinary tumors are also gradually being considered. Generally, the closer the administration route is to the tumor cells, the better the anticancer effect and the lower the side effects. For example, intravesical instillation chemotherapy for bladder malignancies (Griffin and Holzbeierlein, 2013). If these chemotherapy drugs are administered intravenously, they often cause gastrointestinal discomfort, local rash, or hair loss—chemotherapy syndromes (Dreno, 1990). The good lipophilicity of PDEVs compared to traditional chemotherapy drugs makes them more absorbed by mucous membranes, thus allowing appropriate reduction of PDEV concentration to avoid potential biological risks from high concentrations (Xu et al., 2023). However, before clinical application of PDEVs, the clinical administration concentration of PDEVs needs attention. For example, it is very difficult to extrapolate from *in vitro* studies to clinical doses suitable for human research, and these factors inevitably vary significantly depending on the administration route (Livingstone et al., 2019). Prerequisites for *in vivo* experiments require sufficient understanding of the bioavailability of PDEVs *in vivo* (Weaver et al., 2017). One option is to attempt treatment by orally administering PDEVs similar to dietary supplements (Cao et al., 2019). But naturally plant-derived PDEVs often cannot withstand decomposition by gastric acid, and in the gastrointestinal environment, some loaded bioactive molecules such as proteins and nucleotides may be inactivated prematurely. Another option is to use genome editing technology to modify PDEVs to adapt to different microenvironments, such as alkaline environments—intravesical, or acidic environments—intragastric, to be able to provide increased bioactivity of PDEVs while maintaining relative stability of effective components (Mithen et al., 2003). In practice, studying the utility of PDEVs in genitourinary malignancies also requires prior knowledge of the levels of bioactive substances in related plants. Many plant bioactive substances have been shown to have pleiotropic effects on prostate cancer cells. Epidemiological studies have shown that foods such as *cruciferous vegetables, allium vegetables, tomatoes, red wine, green tea, turmeric*, and *pomegranate* are associated with reduced PCa risk (Livingstone et al., 2019). To understand the role of common edible plants and their derived PDEVs in preventing aggressive PCa, well-designed human intervention studies are needed for further insight.

**FIGURE 4:**
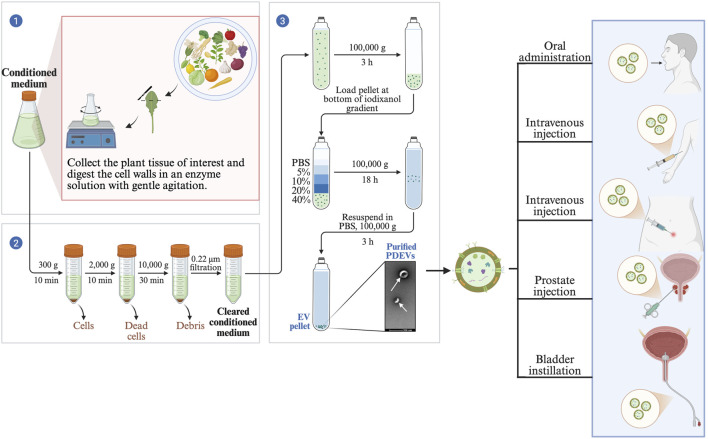
Extraction of PDEVs and potential clinical applications.

Oral administration is considered a potential route for the clinical application of PDEVs (Figure 4). Because oral treatment has many advantages, such as high patient compliance and overcoming toxicity issues (Yang et al., 2020). PDEVs have been used for oral delivery of paclitaxel to improve efficacy and reduce toxicity (Agrawal et al., 2017). It is well known that although the composition of the gut microbiota is relatively stable throughout an individual’s life, the occurrence of disease can induce changes (David et al., 2014). For example, tumor diseases can interfere with intestinal probiotic bacteria affecting the body’s immunity (Tomova et al., 2019). Thus, similar to TCM therapy, regulating the whole body by oral administration of Chinese herbs can also achieve cancer treatment effects (Wang et al., 2020; Zhang and Xiao, 2021). In fact, PDEVs also have effects similar to TCM in maintaining intestinal symbiosis and homeostasis, which may be a potential way to treat genitourinary malignancies (Rome, 2019). Regarding the oral route, acid resistance needs special attention. Studies have found that some plant-derived PDEVs have natural acid resistance. For example, *turmeric*-derived PDEVs maintained intact structure in the gastrointestinal tract, with the only significant change being a shift in surface charge from negative to positive due to hydrogen ion neutralization in gastric acid (Nemidkanam and Chaichanawongsaroj, 2022). It is worth noting that not all PDEVs change surface charge in gastric acid. For tea flower-derived PDEVs, their structure and potential remained unchanged in simulated gastric fluid, simulated small intestinal fluid, and simulated colon fluid. These acid-resistant PDEVs can protect anticancer drugs in the microenvironment until they are taken up by cancer cells. Therefore, these stable PDEVs may be excellent nano-platforms for loading drugs for genitourinary malignancies. A typical example of oral utilization of PDEVs loaded with antitumor drugs is sorafenib. Due to degradation by gastric acid, the oral bioavailability of sorafenib is significantly limited. When sorafenib was encapsulated in kiwi-derived PDEVs, it showed significant stability even under high gastric acid conditions, and absorption rate was also significantly improved (Fang et al., 2023).

Intravenous administration is also considered a preferred route for the clinical application of PDEVs. For example, phosphodiesterase inhibitors (PDEIs) derived from grapefruit cannot cross the placental barrier when administered intravenously, indicating that intravenous PDEIs from specific plant sources do not interfere with pregnancy. This offers significant advantages for treating pregnant women with urogenital malignancies (Wang et al., 2013). Compared to oral administration, intravenous injection allows early accumulation of PDEVs at the tumor site, which may produce stronger pharmacological effects. However, higher levels of complement C3, alanine aminotransferase, and aspartate aminotransferase were detected in mice receiving intravenous injection of these PDEVs compared to the oral route, indicating higher liver toxicity associated with intravenous injection (Chen A. et al., 2022).

PDEVs have been delivered through various *in vivo* models via different routes, such as oral, nasal, intravenous, and intraperitoneal injection. Depending on the primary location of the tumor, the administration route for PDEVs may vary. For example, nasal administration is often used for delivery to the brain; PDEVs delivered intravenously or intraperitoneally tend to enrich in the liver and spleen (Lian et al., 2022). Maintaining gastrointestinal homeostasis is the choice for oral and gastric administration. Therefore, for bladder malignancies, anti-urothelial carcinoma PDEVs can be used for intravesical instillation chemotherapy; for prostate cancer, PDEVs can be injected directly perineally or rectally; and for renal cancer, oral or intravenous administration can be used since most soluble substances are metabolized by the kidneys. Biological barriers can block foreign substances to maintain homeostasis but also hinder drugs from reaching the lesion. For example, the blood-testis barrier impedes chemotherapy delivery to testicular tumors. Intratesticular injection of lipophilic PDEVs may enable them to penetrate this barrier and deliver anticancer drugs, thereby enhancing therapeutic efficacy (Zh et al., 2016). Therefore, choosing the appropriate administration route to improve conditions for PDEVs to approach GU tumor cells will enhance the bioavailability of PDEVs in GU cancers and reduce their toxic effects.

## Engineered plant-derived extracellular vesicles for targeted therapy of genitourinary tumors

With global aging, the incidence of genitourinary (GU) cancers is increasing year by year (Koti et al., 2023). Most patients diagnosed with GU cancers are often over 65 years old (Bukavina et al., 2022), and elderly patients have more significant recurrence rates, drug resistance, and poor metastasis prognosis (Zhang et al., 2022). Although medical technology has made great progress in recent years and the survival rate of GU cancer is improving annually, clinical outcomes for drug-resistant and metastatic GU cancer remain unsatisfactory (Evans et al., 2024). Cancer is an evolving disease with unpredictable outcomes. Although traditional chemotherapy can significantly inhibit cancer, lack of specificity and poor bioavailability remain major problems in cancer treatment, for example, in childhood reproductive system tumors (Sklar and LaQuaglia, 2000). Although chemotherapy is one of the main treatments for tumors, it also has obvious limitations, including low delivery efficiency, tissue resistance, and insufficient drug targeting (Dash et al., 2015). At the same time, they can also cause serious side effects including cardiac, neurological, gastrointestinal, and renal toxicity, bone marrow suppression, stomatitis, and hair loss, which compel us to find methods that can selectively target cancer cells and minimize treatment-related toxicity. Due to the inherent biocompatibility and cell targeting of PDEVs and their regulatory effects on cancer cells, efficient encapsulation of drugs and genetic materials maintains drug stability, and modification with specific targeting markers achieves precise anticancer effects *in vitro* and *in vivo* (Figure 3). For example, common genitourinary system chemotherapy drugs include anthracyclines (e.g., doxorubicin and daunorubicin), anti-pyrimidine nucleotide metabolism drugs (gemcitabine), paclitaxel, etc. Advances in engineering technology make the customization of PDEVs possible, such as surface modification to increase affinity for specific tumor markers, improving PDEV targeting efficiency and therapeutic drug loading capacity while enhancing anticancer effects. This tumor-targeting mode is more effective for heterogeneous tumors because it does not rely on unique or overexpressed cancer-associated antigens on the surface of tumor cell populations, which is a prerequisite for antibody-mediated targeted drug delivery (Huang et al., 2023). Novel immunotherapies have significantly changed the treatment landscape of GU cancers by modulating the immune system to fight tumor cells. Although these therapies are effective, not all GU tumors respond to immunotherapy (Zou et al., 2016). This suggests the need to focus on new methods to overcome immunotherapy resistance. Since some PDEVs have innate immunomodulatory effects and can load various types of immunomodulators, such as small molecule drugs, nucleic acids, and proteins, they may be ideal candidates for overcoming cancer immunotherapy resistance, including in GU cancers (Kameli et al., 2021; Karamanidou and Tsouknidas, 2021). In the early stage of GU cancer, the interaction of genetic and microenvironmental factors enables cancer to resist immune attack, from disruptions in immune pathways to changes in microbiome effects leading to cancer resistance to immunotherapy (Said and Ibrahim, 2023). Engineering modified PDEVs to load signaling molecules missing in immune pathways may help improve sensitivity to immunotherapy in early-stage cancer. In the middle stage of GU cancer, alterations in antigen processing and presentation help GU tumor cells escape immune killing, especially in bladder and prostate cancer (D'Urso et al., 1991). For prostate cancer, the gut microbiota-mediated immunomodulation process is also a potential way for tumor cells to evade immunity. For example, prostate cancer-specific therapies (androgen receptor-targeted therapy) have effects on the gastrointestinal microbiota. These microbiota are not only affected by such treatments but can also promote resistance to these therapies (Burns et al., 2019; Golombos et al., 2018). This mechanism may involve gut bacteria in cancer patients directly regulating the body’s anticancer immune defense pathways (Routy et al., 2018). And PDEVs may play an important role in maintaining gut microbiota homeostasis (Rufino-Ramos et al., 2017). Natural Chinese herbs have been confirmed to maintain intestinal homeostasis by regulating gut microbiota, and their derived PDEVs may have similar effects. In the late stage of GU cancer, adaptive changes become an important factor in cancer immune tolerance, mainly involving dynamic modifications of the tumor, i.e., the tumor resisting immune system attacks during treatment, such as the emergence of an immunosuppressive tumor microenvironment formed by the synergistic action of IL-8 and VEGF (Kim, 2020; Martin et al., 2009). At the same time, GU tumors can also secrete various immunomodulatory substances, altering the local immune state and leading to immune tolerance (Cerezo-Wallis et al., 2020). Interestingly, specific plant-derived PDEVs can participate in intercellular communication in plants as a means of regulating innate immunity (Rutter and Innes). Thus, PDEVs may also act as immune enhancers to alter the tumor microenvironment, affect the expression of IL-8 and VEGF or other immune factors, and improve the sensitivity to immune drugs in the late stage of GU cancer. In summary, for GU cancer, PDEV therapy is a promising and feasible alternative. First, this therapy can change the phenotype of target cells by transferring nucleic acids. By introducing tumor suppressor genes, regulating oncogenes, inhibiting angiogenesis, directly or indirectly inducing cancer cell apoptosis, and activating the immune system to better attack cancer cells (Jayasinghe et al., 2021). Second, this therapy can use targeting ligands to guide PDEVs to the tumor microenvironment to promote changes in existing specific stimuli (Jayasinghe et al., 2021). For example, research designed enzymatic PDEVs expressing native GPI-anchored interleukin-2, which could improve anticancer efficiency by targeted delivery of immunosuppressive effects on the tumor microenvironment (Zhang et al., 2010). However, before engineering PDEVs for the treatment of GU cancer, some issues may need consideration. Several key factors such as plant source, isolation and extraction methods, mechanisms of action, and drug loading capacity are related to the efficacy and safety of PDEVs. Whether the delivered drug is natural or synthetic, the practicality of PDEVs needs to be further established compared to conventional medical interventions to optimize reproducibility, dose toxicity, specificity, structural integrity, and cost-effectiveness. In this regard, a clinical trial (NCT01294072) was initiated in 2011 at the University of Louisville to evaluate the ability of plant-derived PDEVs to promote curcumin delivery to normal and cancerous colon tissues.

## Discussion and outlook

Although many substantial advances have been made in the treatment of GU malignancies over the past few decades, gradually improving the survival and quality of life of GU malignancy patients (Evans et al., 2024), the development of complementary and alternative options for GU cancers is still anticipated. The use of TCM regimens in GU cancer patients is becoming increasingly popular and advocated, especially for prostate cancer (Philippou et al., 2013). In clinical practice, for GU malignancies, the international community is also beginning to recommend integrated traditional Chinese and Western medicine treatment models, believing that this will help improve the adverse reactions of anticancer drugs and patient survival (Philippou et al., 2013). Related studies confirm this. For hormone-sensitive prostate cancer patients, combining TCM can delay the development of androgen-independent prostate cancer, reduce PSA levels, and improve patient 5-year survival rates, without affecting the process of androgen deprivation therapy (Kong et al., 2023). For urothelial carcinoma, milk thistle extract can be used via intravesical instillation chemotherapy to reduce the risk of recurrence in superficial bladder cancer, especially after transurethral resection (Zeng et al., 2011). But in fact, due to the complexity of their components, Chinese herbs often bring potential liver and kidney toxicity and trigger allergic reactions, and single extracts from plants also have systemic toxicity similar to chemotherapy drugs, such as intravenous use of colchicine (Rubicondo et al., 2025). With the development of nanomedical technology, PDEVs represent an innovative frontier in oncology research, combining the advantages of natural anticancer properties, biocompatibility, and precise drug delivery, making it increasingly apparent that PDEVs can change the cancer treatment paradigm. By leveraging the uniqueness of PDEVs, the side effects of traditional anticancer therapies can be greatly reduced, and anticancer efficacy improved. Of course, the path to clinical application is still filled with a series of challenges. For example, environmental conditions can seriously affect the stability and bioactivity of PDEVs. The intrinsic properties of isolated PDEVs can change with variations in certain physical parameters, including pH, temperature, and other processing factors (Kim et al., 2022; Kim and Kim, 2022). Additionally, plant pathogen invasion often infects and damages plant tissues, and plant-derived PDEVs may contain molecules associated with plant virus infections (Stanly et al., 2019), which could pose potential biohazards. Strict screening of PDEV donors may help avoid this risk, such as using organic plants, seasonal plants, and non-genetically modified plants. PDEVs isolated from different parts of plants may also have substantial differences in intrinsic therapeutic activity or drug delivery capacity. Given the wide variety and abundance of medicinal plants available, priority should be given to isolating PDEVs from medicinal plants with anti-GU cancer properties for early research. In fact, the EU has classified PDEVs as herbal medicinal products, defined as “any medicinal product exclusively containing as active ingredients one or more herbal substances or one or more herbal preparations, or one or more such herbal substances in combination with one or more such herbal preparations” (Kameli et al., 2021). In summary, as natural “nano factories,” PDEVs provide a cost-effective, sustainable, and renewable type of medical resource (Iravani and Varma, 2019). Before applying PDEVs for the treatment of GU cancers, some challenges still need consideration: the mechanisms of PDEV formation and release are unclear; there is no unified naming paradigm; accurate biomarkers are lacking; preservation and transportation technologies need breakthroughs; and the distribution and metabolism mechanisms *in vivo* are vague. At the same time, there is an urgent need to develop more economical and time-efficient extraction methods to adapt to large-scale production. In summary, the application of PDEVs is not limited to changing GU cancer treatment by affecting key biological processes such as GU tumor cell proliferation, apoptosis, and metastasis but can also be used as complementary therapy for antitumor drugs to reduce inflammation, tissue damage, and gut microbiota disorders during treatment (Yang et al., 2023). In new immunotherapies for GU cancer, perhaps PDEVs can help us understand more about immune resistance mechanisms and enhance the therapeutic effect for patients resistant to current GU cancer immunotherapy regimens.

## Conclusion

In genitourinary tumors, alternative and complementary therapies using TCM have been widely accepted. Since the anticancer active components they contain can be selectively incorporated into their derived extracellular vesicles, their auxiliary anticancer effects are being gradually confirmed. The unique properties of plant-derived PDEVs, such as low immunogenicity, low tumorigenic risk, modifiable targeting, loadability, and relatively low cost, demonstrate great anticancer potential in the treatment of genitourinary tumors, which may be the future of nanotherapy for genitourinary tumors. Currently, research on PDEVs for treating genitourinary tumors is limited, and there is still a need to actively clarify their anticancer mechanisms in genitourinary tumors and to find parent plants with significant anticancer activity. In summary, specific PDEVs can serve as alternative and complementary therapies for genitourinary tumors.
