# Correction: Plant-derived extracellular vesicles: an alternative and complementary therapeutic approach for genitourinary tumors

**DOI:** 10.3389/fcell.2025.1741627

**Published:** 2025-11-25

**Authors:** Yuqing Huang, Yunmeng Zhang, Kecheng Lou, Shangzhi Feng, Guoqiang Feng

**Affiliations:** 1 Department of Urology, Jiujiang University Clinic College/Hospital, Jiujiang, Jiangxi, China; 2 Department of Anesthesiology, Jiujiang University Clinic College/Hospital, Jiujiang, Jiangxi, China; 3 Department of Urology, Lanxi People’s Hospital, Jinhua, Zhejiang, China; 4 Department of Rehabilitation, Jiujiang University Clinic College/Hospital, Jiujiang, Jiangxi, China

**Keywords:** plant-derived extracellular vesicles, genitourinary tumors, tumor drug resistance, adjuvant anticancer drugs, engineered extracellular vesicles

There was a mistake in Figure 4 as published. The image was accidentally replaced with another image. The corrected Figure 4 appears below:

The original article has been updated.

**FIGURE 4:**
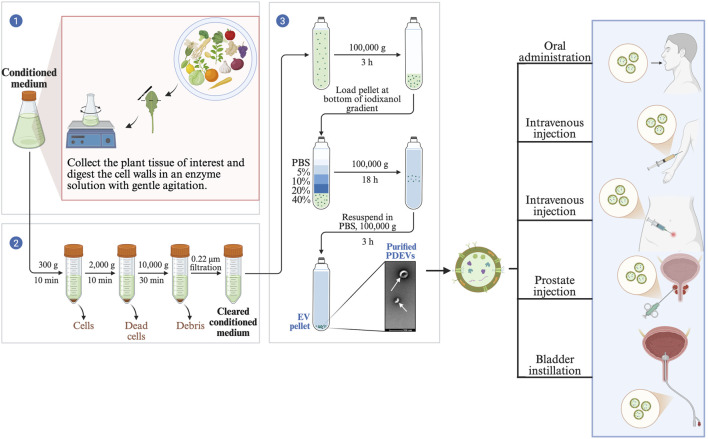
Extraction of PDEVs and potential clinical applications.

